# Fabrication of Size-Controlled Carbon Dots with Biofilm-Disrupting Activity for Antibacterial Applications

**DOI:** 10.3390/ijms27104159

**Published:** 2026-05-07

**Authors:** Yu-Xin Qian, Ming Yu, Ze-Kun Chen, Yue Shen, Lei Tang, Ke-Wu Zeng, Peng-Fei Tu

**Affiliations:** 1 School of Pharmaceutical Sciences, Guizhou Medical University, Guiyang 550014, China; qianyuxin0210@163.com; 2 State Key Laboratory of Natural and Biomimetic Drugs, School of Pharmaceutical Sciences, Peking University, Beijing 100191, China; yming820@bjmu.edu.cn (M.Y.); czkqust@163.com (Z.-K.C.); shenyue0506@126.com (Y.S.); pengfeitu@bjmu.edu.cn (P.-F.T.); 3 Department of Integration of Chinese and Western Medicine, School of Basic Medical Sciences, Peking University, Beijing 100191, China

**Keywords:** carbon dots, particle size, physicochemical properties, antibacterial activity, biofilm disruption

## Abstract

Carbon dots (CDs) have demonstrated broad-spectrum biological activity, with particle size considered a key determinant of their biological efficacy. However, the interrelationships among size, structure, and function remain unclear. To address this, we synthesized CDs under identical hydrothermal protocols and separated them into four size fractions (NPDCDs_1_-NPDCDs_4_), to directly investigate how particle size influences physicochemical and antibacterial properties. The four fractions exhibited distinct optical and structural properties: NPDCDs_1_ (3.2 nm) emitted at 510 nm with the highest C-O content; NPDCDs_2_ (2.2 nm) emitted at 510 nm with high C-C/C=C content; NPDCDs_3_ (2.1 nm) showed red-shifted emission at 570 nm and the highest C=C ratio; NPDCDs_4_ (1.9 nm) displayed the most red-shifted emission at 580 nm (λex = 380 nm) with the highest C=O content. Notably, NPDCDs_1_ demonstrated excellent biocompatibility and potent antibacterial activity, primarily through efficient disruption of bacterial biofilms, possibly due to its high C-O content and appropriate particle size. Thus, particle size modulated biological function via corresponding changes in structural and surface chemical properties. These findings clarify that particle size critically influences both the physicochemical properties and antibacterial activity of CDs, providing an empirical foundation for the rational design of highly efficient and low-toxicity carbon-based antimicrobial materials.

## 1. Introduction

The worldwide spread of antibiotic resistance has created an urgent need for next-generation antimicrobials that canould supersede conventional antibiotics. Nano-materials showed considerable promise as anti-infective therapeutics [1]. Carbon dots (CDs) are an emerging class of carbon-based nanomaterials, typically smaller than 10 nm in size with a quasi-spherical morphology. CDs have gained significant attention in antibacterial nanomaterial research owing to their unique physicochemical properties and superior biocompatibility, particularly their potent ROS generation capability and facile surface modifiability [2,3]. Recent studies have demonstrated that CDs inhibit bacterial growth by generating ROS and disrupting biofilm architecture [4,5]. This unique mechanism provides a novel therapeutic strategy to overcome antibiotic resistance. For instance, copper-doped CDs derived from ciprofloxacin mediated potent electrostatic disruption of bacterial membranes, thereby eliminating *Escherichia coli* (*E. coli*) and *Staphylococcus aureus* (*S. aureus*) with 99.9% efficiency at low concentrations [6]. Moreover, CDs with a core–shell architecture were able to specifically penetrate the dense peptidoglycan layer of Gram-positive bacteria (e.g., *S. aureus*), thereby disrupting biofilms and subsequently exerting bactericidal effects [7]. A critical prerequisite for translating carbon dots from the laboratory to clinical applications lies in ensuring their biosafety toward human cells and tissues, particularly through the systematic evaluation of their cytotoxicity toward mammalian cells and their hemocompatibility. Only by establishing a well-defined safe exposure window while preserving potent antibacterial activity can irreversible host damage be avoided during anti-infective therapy. Thus, CDs with favorable biocompatibility and reactive ROS generation capability can exhibit synergistic mechanisms that are difficult for bacteria to evade, demonstrating outstanding potential as effective alternatives to conventional antibiotics against drug-resistant bacterial infections.

However, most currently reported carbon dots (CDs) are not individual molecules but are instead complex blends containing small molecules, oligomers, and particles ranging from the nano- to micron scale. The presence of impurities significantly affects the physicochemical properties of CD-based mixtures, complicating the resolution of important mechanistic questions [8,9]. Therefore, rigorous and sequential purification is essential to obtain CDs with consistent functionality and well-defined structures [10,11]. Previous studies indicate that, while dialysis effectively removes small-molecule impurities, the application is limited by substantial drawbacks, including prolonged processing durations, elevated consumable costs, and an inability to retain byproducts similar in size to CDs [12]. In contrast, column chromatography allows for the polarity- or size-dependent fractionation of CDs into distinct luminescent subpopulations, yet it demands substantial labor and solvent consumption [13]. Overall, the high complexity of CD mixtures makes purification a significant technical challenge, leading to the absence of standardized purification protocols for obtaining well-defined, uniform carbon dots.

CDs derived from aniline precursors (e.g., o-phenylenediamine or p-phenylenediamine) exhibit broad-spectrum antibacterial activity [13,14,15]. Moreover, amino-functionalization enhances electrostatic binding to bacterial membranes, reducing ROS diffusion distance and boosting bactericidal efficiency [16]. Additionally, the incorporation of CDs into biopolymer matrices enables their application in antibacterial coatings and dressings. To enhance antibacterial efficacy, researchers have employed surface modification strategies including quaternization, metal coordination, and heteroatom doping to increase the ROS-generating capacity of CDs [17]. Consequently, precise control over the intrinsic properties of CDs is essential for optimizing such applications. Currently, research efforts are primarily directed along two strategic paths: (1) the precise regulation of precursor composition during synthesis to preserve optimal sp^2^-hybridized domains, and (2) the use of size-selective purification techniques to exploit quantum confinement effects for tunable emission properties [18]. However, these preliminary investigations have primarily focused on surface modifications, while the critical effects of particle size and chemical composition on antibacterial efficacy remain to be systematically investigated.

In this study, we aimed to elucidate the size-dependent optical properties and antibacterial activities of CDs. Crude CDs featuring distinct sp^2^ conjugated domains and active N/O sites characteristic of CD structures were synthesized via a one-step hydrothermal method. Then, four CDs with distinct sizes were obtained by combining polarity-based and size-based separation techniques. The core structures and surface compositions of the CDs were characterized to reveal distinct correlations between the structural features, spectroscopic properties, size-dependent antibacterial activities, the cytotoxic effects, and- blood compatibility [19,20]. In summary, this work systematically clarified the influence of the particle sizes of CDs on optical characteristics and antibacterial efficacy, thereby providing valuable insights for the rational design of multifunctional antibacterial nanomaterials.

## 2. Results

### 2.1. Synthesis and Characterization of CDs

**Figure 1:**
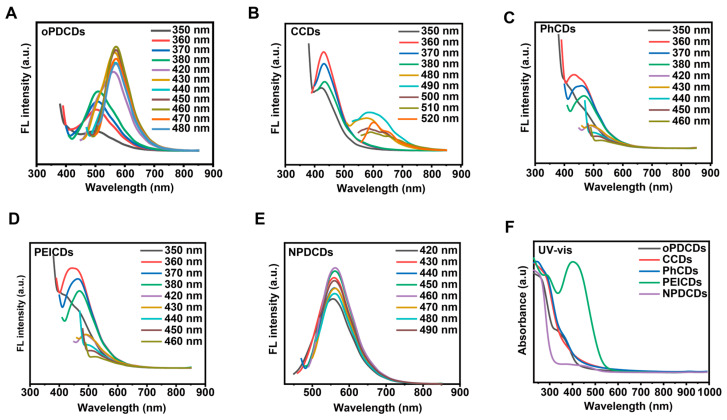
Optical characterization of oPDCDs, CCDs, PhCDs, PEICDs, and NPDCDs. (**A**) Emission spectrum of oPDCDs. (**B**) Emission spectrum of CCDs. (**C**) Emission spectrum of PhCDs. (**D**) Emission spectrum of PEICDs. (**E**) Emission spectrum of NPDCDs. (**F**) UV–vis absorption spectra of the five carbon dots.

During the preparation of carbon dots via the hydrothermal method, PEI and sodium citrate undergo a dehydration condensation reaction to form amide bonds, thereby linking the two together. The mixture continuously undergoes dehydration, crosslinking, and carbonization to form a carbon core. The fluorescence properties of carbon dots are jointly determined by the internal conjugated carbon core and the surface states. Meanwhile, the surface functional groups of carbon dots also play a decisive role in their physicochemical properties and biological activities. Therefore, this study employed a controlled-variable approach to synthesize target carbon dots and four control carbon dots (as described in Section 4.2): CCDs (4-nitro-o-phenylenediamine, polyethyleneimine, sodium citrate), PhCDs (polyethyleneimine, 2,3-diaminophenazine), PEICDs (4-nitro-o-phenylenediamine, polyethyleneimine), and oPDCDs (o-phenylenediamine, polyethyleneimine, sodium citrate, 2,3-diaminophenazine), in order to investigate the necessity of the four precursors for NPDCDs. PEI and sodium citrate serve as the primary carbon sources for synthesizing carbon dots; they form carbon dots through condensation reactions and construct the framework of the carbon dots [21,22,23,24]. 4-Nitro-o-phenylenediamine and 2,3-diaminophenazine participate in the construction of the π-conjugated structure of the carbon core during the hydrothermal carbonization process, thereby constituting an important source of fluorescence for the carbon dots [25,26,27,28]. They are the key molecular fluorophores that endow NPDCDs with strong fluorescence emission. In this study, the fluorescence spectra and UV–vis absorption spectra of the five carbon dots were characterized (Figure 1). The results showed that CCDs and PhCDs, lacking 2,3-diaminophenazine, exhibited no visible fluorescence. In contrast, PEICDs with abundant amino groups displayed strong UV absorption at 410 nm and a red-shifted fluorescence excitation peak at 570 nm. Furthermore, the incorporation of sodium citrate as a co-precursor introduced a substantial density of -COO- and -OH groups onto the surface of the CDs, thereby endowing the resulting NPDCDs with excellent water solubility [17]. Therefore, 4-nitro-o-phenylenediamine, polyethyleneimine, sodium citrate, and 2,3-diaminophenazine were uniformly adopted as raw materials to prepare NPDCDs via a hydrothermal method as the initial CDs, ensuring consistency in all subsequent syntheses of CD protocols.

### 2.2. Synthesis and Characterization of Size-Varied NPDCDs

Given the absence of a universal purification standard, we employed a combined strategy of filtration, polarity, dialysis, and size-based gel column chromatography to separate the as-synthesized NPDCDs [29]. Among these, size-based gel column chromatography was performed using Sephadex G-25, a cross-linked dextran gel that separates molecules according to their hydrodynamic volume: larger molecules elute first, as they are excluded from the internal pores, while smaller molecules are retained longer due to their diffusion into the porous network [30,31,32]. Fractions were monitored via thin-layer chromatography and collected sequentially, yielding four distinct samples: NPDCDs_1_, NPDCDs_2_, NPDCDs_3_, and NPDCDs_4_ (as described in Section 4.3). The properties of these fractions were investigated to elucidate the pathways toward the development of high-quality, stable CDs.

**Figure 2:**
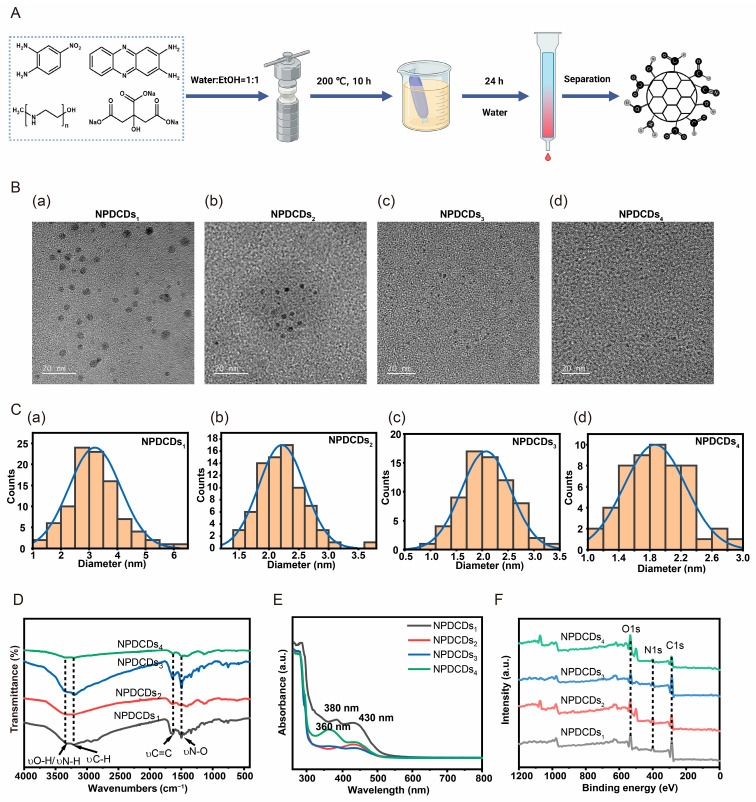
Characterization of NPDCD fractions. (**A**) Schematic illustration of the synthesis and purification of CDs. (**B**) HRTEM images and corresponding size distribution histograms of (**a**) NPDCDs_1_, (**b**) NPDCDs_2_, (**c**) NPDCDs_3_, and (**d**) NPDCDs_4_. (**C**) Statistical analysis of particle size distributions for (**a**) NPDCDs_1_, (**b**) NPDCDs_2_, (**c**) NPDCDs_3_, and (**d**) NPDCDs_4_. (**D**) FT-IR spectra of the four NPDCD fractions. (**E**) UV–vis absorption spectra of the four NPDCD fractions. (**F**) XPS spectra of the four NPDCD fractions.

TEM characterization (Figure 2B,C) revealed that all obtained NPDCD fractions were monodisperse and quasi-spherical. The mean diameters of these CDs were determined to be 3.20 nm, 2.21 nm, 2.08 nm, and 1.90 nm for components 1 to 4, respectively. High-resolution images revealed well-defined crystalline lattices with interplanar spacings of 0.190 nm, 0.203 nm, 0.207 nm, and 0.186 nm. The progressive decrease in size from NPDCDs_1_ to NPDCDs_4_ was consistent with the elution profile observed during fractionation on a Sephadex gel column with ultrapure water, where larger particles eluted first and smaller particles last.

FT-IR spectroscopy was employed to elucidate the chemical composition and functional groups of the NPDCDs (Figure 2D). The FT-IR spectrum of NPDCDs_1_ exhibited a doublet at 3324 cm^−1^ and 3214 cm^−1^ ascribed to the anti-symmetric and symmetric stretching modes of N-H or O-H bonds, while the band at 1632 cm^−1^ corresponded to the in-plane bending vibration of N-H. The signals at 2936 cm^−1^ and 1456 cm^−1^ indicated aliphatic C-H stretching and deformation, respectively. The absorption at 1658 cm^−1^, 1499 cm^−1^, and 1223 cm^−1^ was assigned to C=O, C=C, and C-O stretching vibrations, while the band at 1429 cm^−1^ arose from the in-plane bending of O-H. NPDCDs_2_ exhibited a broad, intense peak centered at 3191 cm^−1^, characteristic of hydrogen-bonded -OH. The spectrum of NPDCDs_3_ was similar to that of the first two fractions, with a pronounced C=C stretching band at 1495 cm^−1^. NPDCDs_4_ displayed a well-resolved doublet at 3351 cm^−1^ and 3214 cm^−1^, confirming the presence of primary amine and hydroxyl functionalities [33].

The optical properties of the NPDCDs in aqueous suspension were examined by UV–vis spectroscopy (Figure 2E). All four fractions of CDs exhibited three distinct maxima at approximately 360, 380, and 430 nm. The 360 nm band originated from the π→π* transition of C=C domains, the peak at 380 nm was attributed to the π→π* transition of C=N linkages, and the 430 nm absorption corresponded to the n→π* transition of C=N bonds. The absorption peaks of NPDCDs_1_ at 380 nm (π→π*) and 430 nm (n→π*) shifted relative to those of NPDCDs_4_, with the former red-shifted by 20 nm, indicating that the decrease in the size of the CDs was accompanied by a narrowing of the sp^2^ domains. The size difference was thus directly imprinted on the electronic structure. Consistent with the TEM images, NPDCDs_1_ exhibited more distinct lattice fringes.

X-ray photoelectron spectroscopy (XPS) was used to determine the elemental composition and chemical bonding states of the four fractions (Figure 2F and Table 1). Firstly, for NPDCDs_1_, the composition was 68.25% C, 9.67% N, and 22.09% O. The high-resolution C 1s spectrum was deconvoluted into five components: C=C (282.45 eV), C-C (284.80 eV), C-O (285.37 eV), and C=O (288.88 eV). Correspondingly, the N 1s spectrum showed pyridinic N (396.58 eV) and graphitic N (399.34 eV), while the O 1s spectrum revealed C=O (530.00 eV) and C-O (532.61 eV). NPDCDs_2_ contained 60.46% C, 16.83% N, and 22.71% O, with C 1s peaks for C=C (282.48 eV), C-C (284.80 eV), C-O (287.31 eV), and C=O (290.56 eV). The peaks for pyridinic N (395.87 eV) and graphitic N (398.73 eV) were assigned to the N 1s spectrum, while the peaks for C=O (530.05 eV) and C-O (532.59 eV) were assigned to the O 1s spectrum. Compared to these two, the high-resolution XPS spectra of NPDCDs_3_ and NPDCDs_4_ exhibited similar functional group peaks (Table 2, Table 3 and Table 4). The combined FT-IR and XPS analysis suggested that, for the larger-sized NPDCDs_1_ and NPDCDs_2_ samples, the carbon skeleton was characterized by relatively high proportions of C–C/C=C and graphitic N, while the oxygen-containing functional groups (especially C=O) were comparatively low. This structural feature is associated with a lower surface polarity. Such characteristics may help reduce non-specific oxidative interactions between the material and mammalian cells, thereby contributing to the observed lower cytotoxicity to some extent. In contrast, the smaller-sized NPDCDs_3_ and NPDCDs_4_ samples exhibited distinct surface chemical features. NPDCDs_4_ showed significantly higher proportions of reactive functional groups such as C-O, C=O, and pyridinic N, indicating abundant polar sites and surface defects. NPDCDs_3_, while having a lower oxygen content, possessed the highest proportion of C=C (sp^2^ carbon) among all samples, reflecting a more extended conjugated structure [34,35].

ζ-potential measurements across a range of pH values (Figure S2) demonstrated that NPDCDs_1_, NPDCDs_2_, and NPDCDs_4_ maintained a negative surface charge at pH values between 3 and 9. In contrast, NPDCDs_3_ exhibited a positive ζ-potential at pH levels below 5, which transitioned to a negative value at pH values above 5. The ζ-potentials of all fractions remained within the range of ±10 mV. Collectively, the data indicated a progressive decrease in the mean particle size from 3.2 nm (NPDCDs_1_) to 1.9 nm (NPDCDs_4_). However, the lattice spacings did not follow a monotonic trend. In contrast, the surface composition evolved consistently across the series: the surface carbon content decreased and the oxygen content increased across the series from NPDCDs_1_ to NPDCDs_4_. Thus, this correlation between size, composition, and separation order highlights the efficacy of the purification process in isolating distinct subpopulations of CDs with tailored properties.

### 2.3. Fluorescence Performance of NPDCDs Size-Varied NPDCDs

**Figure 3:**
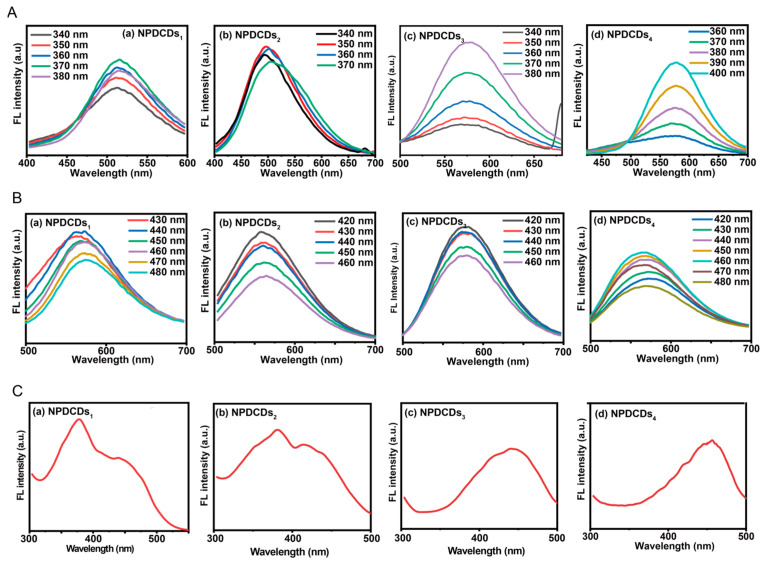
Photoluminescence properties of NPDCDs fractions. (**A**) Emission spectra of (**a**) NPDCDs_1_, (**b**) NPDCDs_2_, (**c**) NPDCDs_3_, and (**d**) NPDCDs_4_ recorded under 340–380 nm excitation. (**B**) Emission spectra of (**a**) NPDCDs_1_, (**b**) NPDCDs_2_, (**c**) NPDCDs_3_, and (**d**) NPDCDs_4_ recorded under 420–460 nm excitation. (**C**) Excitation spectra of (**a**) NPDCDs_1_ (emission at 570 nm), (**b**) NPDCDs_2_ (emission at 560 nm), (**c**) NPDCDs_3_ (emission at 580 nm), and (**d**) NPDCDs_4_ (emission at 570 nm).

The results demonstrated that distinct average particle sizes corresponded to varied physicochemical properties and chemical compositions. Consequently, the optical performance of the four fractionated NPDCDs was systematically investigated (Figure 3). The maximum emissions of NPDCDs_1_ and NPDCDs_2_ were at approximately 510 nm under 340 and 380 nm excitation. Under the same excitation wavelength, the maximum emission wavelength of NPDCDs_3_ was 570 nm, while that of NPDCDs_4_ was 580 nm (Figure 3A). Additionally, the emission maxima of these NPDCDs were at 560–570 nm (NPDCDs_1_), 560–570 nm (NPDCDs_2_), 580 nm (NPDCDs_3_), and 580 nm (NPDCDs_4_) under 420–480 nm excitation (Figure 3B). A red-shift in fluorescence emission was observed as the particle size decreased from 3.2 nm (NPDCDs_1_) to 1.9 nm (NPDCDs_4_).

According to the results presented above, under 340–380 nm excitation, the emission maximum red-shifted with decreasing particle size: NPDCDs_1_ (3.20 nm) and NPDCDs_2_ (2.21 nm) emitted at 510 nm, NPDCDs_3_ (2.08 nm) at 540 nm, and NPDCDs_4_ (1.90 nm) at 580 nm. The red-shift of the NPDCD fraction contradicted the conventional quantum confinement principle that a smaller size leads to blue-shifted emission, indicating that size effects did not dominate the fluorescence mechanism of the present CDs [36]. Complementary characterization data revealed that the reduction in particle size from the early fractions (NPDCDs_1_ and NPDCDs_2_) to the later fractions (NPDCDs_3_ and NPDCDs_4_) was accompanied by an initial increase in graphitization, though NPDCDs_4_ showed a lower sp^2^ carbon content than NPDCDs_3_. This structural evolution enhanced electron delocalization within the sp^2^-conjugated network and consequently narrowed the band gap [37,38]. Concurrently, although NPDCDs_3_ and NPDCDs_4_ both possessed smaller dimensions, their surface chemical characteristics differed. NPDCDs_4_ exhibited significantly high C-O (25.86%) and C=O (10.09%) contents, indicating that the surface states remained pronounced and likely played a dominant role in its fluorescence properties. In contrast, NPDCDs_3_ showed the lowest total oxygen content among all samples (C-O 10.97%, C=O 5.13%) yet possessed the highest sp^2^ carbon (C=C, 27.74%) content, suggesting that its fluorescence may originate more from the carbon core rather than from oxygen-related surface states. These distinctions highlight that, for smaller-sized carbon dots, the contribution of surface states to fluorescence properties is sample-dependent and closely tied to the specific surface chemical composition. Under 340–380 nm excitation, the synergistic interplay between core- and surface-state emissions collectively produced the observed red-shift and fluorescence intensification.

### 2.4. In Vitro Safety Assessment of Size-Varied NPDCDs

**Figure 4:**
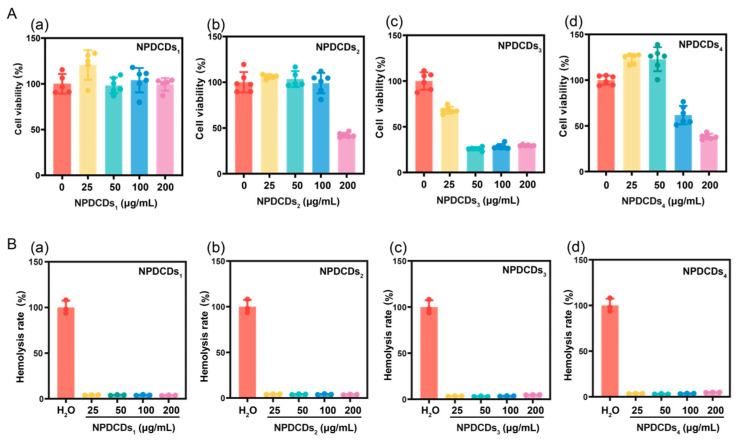
In vitro safety assessment of size-varied NPDCDs. (**A**) Viability of RAW 264.7 cells after 24 h co-incubation with (**a**) NPDCDs_1_, (**b**) NPDCDs_2_, (**c**) NPDCDs_3_, and (**d**) NPDCDs_4_. (**B**) Hemolysis rate of red blood cells after 4 h co-incubation with (**a**) NPDCDs_1_, (**b**) NPDCDs_2_, (**c**) NPDCDs_3_, and (**d**) NPDCDs_4_.

To evaluate the potential applications of the four fractions of CDs for biological studies, we first performed MTT cytotoxicity assays. RAW 264.7, HepG2, and 293T cells were exposed to various concentrations of each NPDCD of distinct dimensions for 24 h, followed by addition of MTT reagent to determine cell viability. As shown in Figure 4A, NPDCDs_1_ demonstrated exceptional biocompatibility (200 μg/mL), while NPDCDs_2_ showed concentration-dependent cytotoxicity (50% viability at 200 μg/mL). In contrast, the cell viability of NPDCDs_3_ and NPDCDs_4_ decreased to about 50%, even at 100 μg/mL. Similar trends were observed in 293T cells (Figure S3). To further evaluate biosafety, we conducted erythrocyte hemolysis assays comparing all four NPDCD fractions. The hemolytic potential of each fraction was assessed by 4 h incubation with fresh erythrocytes under physiological conditions. PBS and ultrapure water served as the negative (0% hemolysis) and positive (100% hemolysis) controls, respectively. Figure 4B shows that none of the four fractions of NPDCDs induced detectable hemolysis. Thus, our data indicated that cytotoxicity increased with decreasing particle size. NPDCDs_1_ (3.2 nm) exhibited excellent biocompatibility up to 200 μg/mL, whereas NPDCDs_2_ (2.21 nm) displayed acceptable hemolysis levels at concentrations ≤ 100 μg/mL. In contrast, NPDCDs_3_ and NPDCDs_4_ induced significant cytotoxic effects at 100 μg/mL, demonstrating clear size-dependent toxicity.

At the molecular level, NPDCDs_1_ and NPDCDs_2_ exhibited larger hydrodynamic diameters and reduced specific surface areas. In contrast, the smaller-sized NPDCDs_3_ and NPDCDs_4_ were more readily taken up by cells and exhibited distinct surface chemistry features—NPDCDs_3_ with the highest sp^2^ carbon content and NPDCDs_4_ with the highest C-O and C=O contents—that contributed to oxidative stress through different mechanisms. Accordingly, the smaller CDs exhibited reduced biocompatibility when compared with NPDCDs_1_ and NPDCDs_2_. Exposure of 293T cells to equal concentrations of these materials yielded consistent results: MTT assays confirmed significantly superior in vitro biocompatibility of one CD subset over the other (Figure S3). This distinction aligns with the classification of these subsets as predominantly quantum-state (nanoparticulate) versus molecular-state (small-molecule) materials, consistent with previous findings.

### 2.5. Antibacterial Activity of NPDCDs_1_

**Figure 5:**
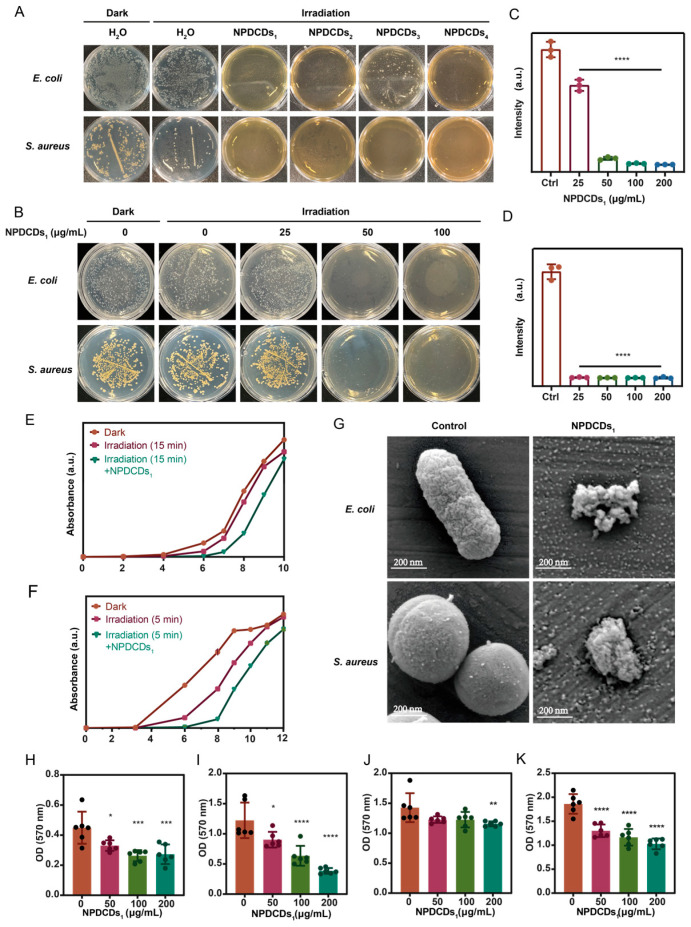
In vitro antibacterial activity of NPDCDs_1_. (**A**) The inhibitory effects of NPDCDs_1_, NPDCDs_2_, NPDCDs_3_, and NPDCDs_4_ on *E. coli* and *S. aureus*. (**B**) Growth of *E. coli* and *S. aureus* on LB agar containing NPDCDs_1_. (**C**) Resazurin reduction assay of *E. coli* after NPDCDs_1_ treatment. (**D**) Resazurin reduction assay of *S. aureus* after NPDCDs_1_ treatment. (**E**) Growth curve (0–10 h) of *E. coli* after NPDCDs_1_ treatment under 450 nm light irradiation. (**F**) Growth curve (0–12 h) of *S. aureus* after NPDCDs_1_ treatment and under 450 nm light irradiation. (**G**) SEM images of bacteria under 450 nm irradiation (left) and bacteria co-incubated with NPDCDs_1_ under 450 nm irradiation (right). (**H**) Crystal violet staining of *E. coli* biofilms after treatment with varying concentrations of NPDCDs_1_. Biofilm biomass was quantified by measuring optical density at 570 nm. (**I**) Crystal violet staining of *S. aureus* biofilms after treatment with varying concentrations of NPDCDs_1_. (**J**) Crystal violet staining was used to assess the clearance effect of NPDCDs_1_ on mature *E. coli* biofilm. (**K**) Crystal violet staining was used to assess the clearance effect of NPDCDs_1_ on mature *S. aureus* biofilm. Bars represent mean ± SEM of at least three independent experiments; data are expressed as mean ± SEM for at least three individual experiments. **** *p*< 0.0001, *** *p* < 0.001, ** *p* < 0.01, * *p* < 0.05.

Next, we evaluated the antibacterial potential of the four size-fractionated CDs. LB solid medium containing each of the NPDCD fractions was prepared, poured into plates while molten, and allowed to solidify. *E. coli* was spread onto the plates, exposed to 450 nm light, and incubated at 37 °C for 12 h. Our colony formation assays revealed a distinct size-dependent antimicrobial effect. The plates treated with NPDCDs1 exhibited the most significant colony reduction, while treatment with NPDCDs_3_ and NPDCDs_4_ resulted in substantially higher colony counts (Figure 5A), demonstrating preserved bacterial viability. Thus, based on the favorable biosafety, aqueous solubility, and potent growth inhibitory effect, NPDCDs_1_ was selected for further investigation.

Antibacterial assays revealed that NPDCDs_1_ exhibited growth suppression of *E. coli* at 25 μg/mL, inhibited *S. aureus* at 50 μg/mL, and demonstrated pronounced antibacterial effects against both strains at 100 μg/mL (Figure 5B). Consistent with the plate assay results, NPDCDs_1_ exhibited concentration-dependent antibacterial effects, with growth inhibition of *E. coli* at 25 μg/mL and *S. aureus* at 50 μg/mL (Figure 5C,D). Moreover, we monitored bacterial growth by optical density at 600 nm (OD_600_). Here is the English translation of the provided paragraph:

Monitoring of bacterial growth curves revealed the temporal dynamic characteristics of the antibacterial effect of NPDCDs_1_. The experiment included a non-illuminated control group, an illumination-only group, and an illumination combined with NPDCDs_1_ group. For *E. coli*, it was observed that the absorbance at 600 nm in the non-illuminated control group began to increase at 4 h, and this trend continued until 10 h, indicating rapid proliferation of *E. coli* during this period. In contrast, 15 min of 450 nm illumination inhibited this rapid proliferation trend, delayed the onset of the rapid growth phase, and suppressed the overall growth trend. The experimental group treated with NPDCDs_1_ exhibited even stronger inhibition of bacterial growth. For *S. aureus*, the experimental results indicated that rapid growth began at approximately 3 h. Even 5 min of 450 nm illumination alone was capable of suppressing its growth rate, while the group treated with NPDCDs_1_ demonstrated the most potent inhibitory effect on the growth of *S. aureus*. Furthermore, it was evident that the inhibitory effects on bacteria, whether induced by illumination alone or by NPDCDs_1_ combined with illumination, were time-limited. After a certain period, the inhibitory effects diminished, and the rate of bacterial proliferation increased (Figure 5E,F). The morphological impact of NPDCDs_1_ on bacterial cells was assessed by SEM. Following standard preparation (glutaraldehyde fixation, dehydration, and sputter-coating), the treated cells exhibited clear signs of damage, including shrinkage and membrane rupture. In sharp contrast, the untreated control cells maintained intact, smooth surfaces (Figure 5G).

We next performed broth microdilution assays to determine the minimum inhibitory concentration (MIC) of NPDCDs_1_ against both *E. coli* and *S. aureus*. The results show that NPDCDs_1_ exhibited an MIC of 200 μg/mL against both strains. These findings are consistent with our previous observations: at 200 μg/mL, the resazurin assay indicated complete inhibition of bacterial metabolic activity, and the colony formation assay showed no visible colonies on agar plates (Figure S4).

Collectively, these results demonstrated strong antibacterial activity for the largest fraction of NPDCDs (NPDCDs_1_) under 450 nm light. This fraction of NPDCDs effectively eliminated both Gram-negative and Gram-positive bacteria. In contrast, smaller CDs showed much weaker activity. These findings reveal a clear link between larger particle size and better antibacterial performance.

As shown in Figure 5H,I, the OD_570_ values measured by crystal violet staining decreased in a dose-dependent manner with increasing concentrations of NPDCDs_1_. Compared with the control group (0 μg/mL), a reduction in OD570 was observed at a concentration of 50 μg/mL; at concentrations of 100 μg/mL and 200 μg/mL, the OD_570_ values further decreased, exhibiting significant differences. These results indicate that NPDCDs_1_ effectively inhibited *E. coli* biofilm formation in a concentration-dependent manner. NPDCDs_1_ also exhibited a dose-dependent inhibitory effect on *S. aureus* biofilm. Compared with the control group, a significant reduction in OD_570_ was observed at 50 μg/mL; at concentrations of 100 μg/mL and 200 μg/mL, the OD_570_ values continued to decline, reaching highly significant levels.

Based on the biofilm inhibition experiments, we further evaluated the ability of NPDCDs_1_ to eradicate pre-formed mature biofilms. *E. coli* and *S. aureus* were cultured in 96-well plates for 48 h to allow mature biofilm formation. The planktonic bacteria were then removed, and fresh medium containing different concentrations of NPDCDs_1_ (0, 50, 100, and 200 μg/mL) was added to the wells for an additional 6 h of treatment. Residual biofilms were quantified using crystal violet staining. The results are shown in Figure 5J,K. Compared with the blank control group, treatment with NPDCDs_1_ reduced the biofilm biomass of both strains to a certain extent. For *E. coli*, the OD570 values in the 50, 100, and 200 μg/mL treatment groups were 1.20, 1.30, and 1.20, respectively, all lower than the control value of 1.40. For *S. aureus*, the OD_570_ values exhibited a dose-dependent decrease. Together, these two sets of experiments confirm that NPDCDs_1_ has a disruptive effect on bacterial biofilms.

## 3. Discussion

In this study, nitrogen-doped photoluminescent carbon dots (NPDCDs) were successfully synthesized via a hydrothermal method, and four distinct size fractions (3.2–1.9 nm) were subsequently isolated through multi-step purification. As the particle size decreased, increased surface oxidation and a fluorescence red-shift from 510 nm to 580 nm were observed, revealing a size-dependent optical behavior that deviates from conventional quantum confinement effects [30]. More importantly, a size-dependent divergence between antibacterial activity and cytotoxicity was identified: the largest fraction (NPDCDs_1_, 3.2 nm) exhibited potent antibacterial efficacy under 450 nm irradiation while maintaining excellent biocompatibility, whereas the smallest fractions (≤2.08 nm) demonstrated cytotoxicity but weaker antibacterial performance. This phenomenon may be attributed to the relatively intact conjugated structure, which helps maintain its physical antibacterial effect against bacteria [28]. Concurrently, we postulate that the larger-sized NPDCDs_1_ fraction, by virtue of its relatively well-preserved aromatic conjugated architecture, may function as a highly efficient photosensitizer upon 450 nm blue-light excitation, thereby generating reactive oxygen species via energy transfer mechanisms to disrupt the extracellular polymeric substances within the biofilm matrix and eradicate the encapsulated bacteria [39,40].

However, the isolated fractions not only exhibited a gradient in particle size but also demonstrated significant differences in surface chemical properties, including the degree of oxidation and the types of nitrogen-containing functional groups. Although this study observed and described these variations, it did not systematically establish the intrinsic links among size, surface chemistry, and biological activity. The current data are insufficient to completely decouple the size effect from the complex surface chemistry, thereby precluding the identification of size as the sole determinant of biological function. Future research should focus on disentangling these interdependent parameters to elucidate how size and surface chemistry synergistically regulate the differential antibacterial activity.

## 4. Materials and Methods

### 4.1. Materials

4-Nitro-o-phenylenediamine was obtained from Mreda (Beijing, China). O-phenylenediamine and 2,3-diaminophenazine were purchased from Macklin (Shanghai, China). Sodium citrate, phenazine, and branched polyethyleneimine (MW ~25,000) were sourced from Aladdin (Shanghai, China). All chemicals were of at least analytical grade, and ultrapure water was used exclusively for preparing solutions and conducting experiments. Dialysis bags with a molecular weight cutoff (MWCO) of 500 Da were obtained from Yuanye (Shanghai, China). Heparin sodium anticoagulant vacuum blood collection tubes were purchased from Yijiajia (Wuhan, Hubei, China). Trypsin (0.25%) and Dulbecco’s Modified Eagle Medium (DMEM, high glucose) were purchased from MacroGene (Beijing, China).

### 4.2. Synthesis of CDs

Initially, NPDCDs were synthesized via a one-step hydrothermal method, using 4-nitro-o-phenylenediamine (1 g), polyethyleneimine (1 g), sodium citrate (1 g), and 2,3-diaminophenazine (0.1 g). These materials were dissolved in a solvent mixture of water and ethanol. The resulting solution was transferred into a Teflon-lined stainless-steel autoclave and heated at 200 °C for 10 h. After cooling to room temperature, the crude mixture was centrifuged at 8000 rpm for 10 min, and this washing process was repeated once. The collected supernatant was then dialyzed against ultrapure water using a 500 Da molecular weight cut-off membrane for 24 h. Finally, the purified solution was lyophilized for 48–72 h to yield solid NPDCD powder.

To investigate the role of each precursor, four additional carbon dots were prepared using the same hydrothermal procedure, with the precursor compositions adjusted as follows (Table 5):

### 4.3. Preparation of Size-Varied NPDCDs

The crude NPDCDs were redissolved in distilled water, sonicated for 20 min, and filtered through a 0.22 μm membrane. To remove unreacted organic starting materials, the product was further washed with a low-polarity solvent, petroleum ether, to eliminate residual aniline derivatives and other organic impurities. The resulting liquid was pre-cooled at −80 °C for 2 h and lyophilized for 72 h to obtain a solid, which was designated as crude product 2. Crude product 2 was dissolved in distilled water at a concentration of 100 mg/mL and sonicated for 20 min. This solution was applied to a Sephadex G-25 column, with pure water used as the eluent. The fractions were collected in the order of elution and designated as NPDCDs_1_, NPDCDs_2_, NPDCDs_3_, and NPDCDs_4_. Each fraction was concentrated by rotary evaporation, pre-cooled at −80 °C for 2 h, and lyophilized to obtain the purified CDs [3,20].

### 4.4. Structural Characterization of NPDCDs

UV–vis absorption spectra of the NPDCD fractions and other CDs were scanned between 300 nm and 1000 nm using a UV–vis spectrophotometer (Thermo 50 UV-vis, Waltham, MA, USA). Zeta potential (ζ-potential) analysis was performed using a Zetasizer (Malvern Zetasizer nano ZSP, Malvern, Worcs, UK) to determine the surface charge on the NPDCDs. The samples were dispersed in buffer solutions with different pH values and subjected to ultrasonication for 20 min to ensure homogeneity. All measurements were performed across a pH range of 3.0–9.0 at a constant temperature of 25 °C. Within the instrument, the colloidal suspension was illuminated by a laser beam, and light scattering by the particles was analyzed to assess surface charge and colloidal stability. Functional groups of the NPDCDs were identified by Fourier transform infrared (FT-IR) spectroscopy (Thermo Nicolet iS50, Waltham, MA, USA), with spectra recorded over 4000–400 cm^−1^. Sample morphology was examined using a high-resolution transmission electron microscope (JEOL JEM-F200, Tokyo, Japan). For TEM observation, each fraction of NPDCDs was dissolved in absolute ethanol and ultrasonicated for 20 min. A small aliquot of the resulting dispersion was then drop-cast onto a copper grid and allowed to dry at room temperature. Fluorescence spectra were recorded on a fluorescence spectrophotometer (Agilent Eclipse, Santa Clara, CA, USA). Excitation wavelengths of 340–380 nm and 420–460 nm were used. The elemental composition and chemical states were determined by X-ray photoelectron spectroscopy (Thermo Fisher ESCALAB 250Xi, Waltham, MA, USA). The absorbance was measured using a microplate reader (Tecan F50, Männedorf, Switzerland).

### 4.5. Cell Culture

The cells used in this study were obtained from the following sources: HepG2 cells were obtained from the Peking Union Medical College Cell Bank (Beijing, China), RAW264.7 cells were obtained from Procell Life Science & Technology (Wuhan, China), and 293T cells were obtained from the National Collection of Authenticated Cell Cultures (Shanghai, China). The cells were authenticated by STR profiling and confirmed to match the ATCC database. Mycoplasma testing was negative. The STR report is uploaded as Supplementary Information. All cells were cultured in DMEM supplemented with 10% (*v*/*v*) fetal bovine serum, 100 U/mL penicillin, and 100 μg/mL streptomycin at 37 °C in a humidified atmosphere containing 5% CO_2_.

### 4.6. In Vitro Cytotoxicity Assay

Cell viability was assessed using an MTT (3-(4,5-dimethylthiazol-2-yl)-2,5- diphenyltetrazolium bromide) assay. Briefly, cells were seeded into 96-well plates at a density of 0.8 × 10^4^ cells per well and incubated at 37 °C under 5% CO_2_ for 12 h to allow adherence. The culture medium was then replaced with fresh medium containing different concentrations of NPDCDs (0, 25, 50, 100, and 200 μg/mL), and the cells were incubated for another 24 h. After this incubation, the medium was removed and replaced with fresh medium containing 0.5 mg/mL MTT, followed by a further 2 h incubation. After washing with phosphate-buffered saline (PBS), 100 μL of dimethyl sulfoxide (DMSO) was added to each well to dissolve the formazan crystals. The plate was shaken for 10 min, and the absorbance was measured at 490 nm using an F50 absorbance microplate reader (Tecan F50, Männedorf, Switzerland).

### 4.7. Hemolysis Assay

For the hemolysis assay, 3 mL of anticoagulated mouse blood was centrifuged at 3000 rpm for 10 min. The harvested red blood cells (RBCs) were washed and resuspended in 0.9% saline to achieve a 1:24 (*v*/*v*) dilution. Then, 200 μL of the RBC suspension was mixed with an equal volume (200 μL) of solutions containing CDs at various concentrations (0, 25, 50, and 100 μg/mL). The mixtures were incubated at 37 °C for 4 h, with saline and ultrapure water serving as the negative and positive controls, respectively. After centrifugation at 3000 rpm, 100 μL of the supernatant was transferred to a 96-well plate, and the absorbance was measured at 545 nm using an F50 absorbance microplate reader (Tecan F50, Männedorf, Switzerland). The morphology of the red blood cells was also observed under an optical microscope.

### 4.8. Bacterial Strains and Culture

The *E. coli* and *S. aureus* strains used in this study were kindly provided by Dr. Fei Cao’s research group at Hebei University (Baoding, China). Species identity was confirmed by 16S rRNA gene sequencing followed by BLASTn analysis against the NCBI nucleotide database. The 16S rRNA sequences exhibited 100% identity and 100% query coverage to *E. coli* and *S. aureus*, respectively. The 16S rRNA sequences are provided in the Supplementary Information (Tables S1 and S2).

For bacterial culture, frozen stock cultures were streaked onto Luria–Bertani (LB) agar plates and incubated at 37 °C for 18–24 h. Single colonies were then inoculated into LB broth and grown overnight at 37 °C with shaking at 180 rpm. The overnight cultures were diluted in fresh LB medium to the desired concentration for subsequent experiments.

### 4.9. Plate Inhibition Assay and Bacterial Growth Curve Analysis

Glycerol stocks of *E. coli* and *S. aureus* were streaked onto LB agar plates and incubated at 37 °C for 24 h. A single colony from each strain was then transferred into fresh LB broth and incubated under the same conditions until the late-logarithmic growth phase was reached (approximately 8 h). LB agar (40 g/L) was sterilized by autoclaving and cooled to approximately 50 °C. NPDCDs were then added to achieve final concentrations of 0, 25, 50, 100, and 200 μg/mL. The supplemented medium was poured into sterile Petri dishes (approximately 20 mL per dish) and allowed to solidify at room temperature.

Subsequently, 100 μL of logarithmic-phase bacterial suspension (approximately 10^6^ CFU/mL) was evenly spread onto the agar surface. The plates were exposed to 450 nm LED light for 15 min (*E. coli*) or 5 min (*S. aureus*) and then incubated at 37 °C for 12 h (the light source used in this study was an LED with a power of 500 W, a wavelength of 450 ± 5 nm, and an irradiation distance of approximately 10 cm). Colony-forming units were enumerated and recorded. Log-phase cultures were diluted to a uniform starting density and allocated to three experimental cohorts: (i) blank control (neither light nor NPDCDs), (ii) light-only control, and (iii) NPDCDs (100 μg/mL) plus light. Following 450 nm illumination, all cultures were incubated at 37 °C with continuous orbital agitation (200 rpm). Optical density at 600 nm (OD_600_) was recorded at 0, 2, 4, 6, 8, 10, and 12 h intervals to construct bacterial growth curves [3].

### 4.10. Determination of the Minimum Inhibitory Concentration (MIC)

The minimum inhibitory concentration (MIC) of NPDCDs_1_ against *E. Coli* and *S. aureus* was determined using the broth microdilution method. Briefly, NPDCDs_1_ was serially diluted in LB medium and then mixed with bacterial suspensions at a 1:1 ratio. Based on preliminary experimental results, the final concentrations of NPDCDs_1_ were set to 100, 200, 300, 400, 600, and 800 μg/mL, while the final bacterial concentration was adjusted to 1 × 10^6^ CFU/mL. The control group consisted of equal volumes of LB medium and bacterial suspension. The total volume per well was maintained at 100 μL (n = 3), and sterile PBS was added to the peripheral wells to prevent evaporation-induced edge effects. The plates were incubated at 37 °C with 5% CO_2_ for 24 h. The MIC was defined as the lowest concentration of NPDCDs_1_ at which the medium remained visibly clear, indicating no bacterial growth.

### 4.11. Bacterial Morphological Analysis

To preliminarily investigate the bactericidal mechanism of NPDCDs_1_, this study employed scanning electron microscopy (SEM, CIQTEK SEM5000X, Hefei, Anhui, China) to examine the effect of NPDCDs on the surface morphology of bacteria. The bacteria were divided into two groups: the blank group received no treatment, while the treatment group was exposed to NPDCDs at a final concentration of 100 μg/mL. The samples were subsequently illuminated at 450 nm for 15 min (*E. coli*) and 5 min (*S. aureus*), respectively. Both bacterial suspensions were incubated at 37 °C for 6 h. Subsequently, an appropriate volume of the bacterial suspension was subjected to centrifugation (4000 rpm for 5 min) and washed twice with PBS. The bacterial pellet was then resuspended in a 2.5% glutaraldehyde fixative solution and fixed overnight at 4 °C. The fixed samples were dehydrated using ethanol, sputter-coated with gold, and subsequently examined under SEM to observe the morphology of *E. coli*. *S. aureus* was processed and prepared for observation using the same methodology, and the morphology was also examined under SEM.

### 4.12. Detection of Bacterial Biofilm Formation by Crystal Violet Staining

To verify the inhibitory effect of NPDCDs_1_ on bacterial biofilm formation, we employed the crystal violet staining method to quantify the biofilm. *E. coli* and *S. aureus* were cultured at 37 °C with shaking at 1000 rpm for approximately 8 h until the logarithmic growth phase was reached. NPDCDs_1_ solutions were prepared in sterile culture medium at final concentrations of 0, 50, 100, and 200 μg/mL. In a 96-well plate, a control group (100 μL bacterial suspension + 100 μL blank medium) and treatment groups (100 μL bacterial suspension + 100 μL NPDCDs_1_ solution at various concentrations) were established, with 6 replicate wells per group. The 96-well plate was exposed to a 450 nm light source for 15 min for *E. coli* or 5 min for *S. aureus*, followed by static incubation at 37 °C for 24 h. After incubation, the culture medium was aspirated, and each well was fixed with 100 μL of 1% methanol at room temperature for 15 min. The methanol was then removed, and the plate was inverted to air-dry. Subsequently, 100 μL of 0.1% crystal violet solution was added to each well and allowed to stain at room temperature for 15 min. The wells were rinsed three times with PBS buffer to remove unbound dye, and the plate was again inverted to air-dry. Finally, 100 μL of 30% acetic acid was added to each well for destaining for 10 min. The absorbance was measured at 570 nm (OD_570_) using a multimode microplate reader, with OD_570_ values being positively correlated with the total biofilm mass.

### 4.13. Statistical Analysis

All experiments were conducted in triplicate to ensure reproducibility. Data were processed using Origin 2021 software and are expressed as mean ± standard deviation (SD). Statistical analysis was performed using a one-way analysis of variance (ANOVA), followed by Student’s *t*-test for multiple comparisons. Differences were considered statistically significant at *p* < 0.05.

## 5. Conclusions

Size-fractionated NPDCDs (3.2–1.9 nm) were systematically investigated. Contrary to a monotonic trend, surface oxidation was not continuously elevated with decreasing particle size; NPDCDs_3_ showed the lowest C-O/C=O content, while NPDCDs_4_ exhibited the highest. A fluorescence red-shift from 510 nm to 580 nm was observed along with these non-monotonic surface chemical changes. A size-dependent toxicity profile was revealed. Negligible cytotoxicity and hemolysis were observed for NPDCDs_1_ (3.2 nm) up to 200 μg/mL, whereas cell viability was reduced to 50% at 100 μg/mL for smaller fractions (≤2.08 nm). Under 450 nm irradiation, the strongest antibacterial activity was demonstrated by NPDCDs_1_. Bacterial cell rupture was evidenced by SEM; diminished efficacy was exhibited by smaller fractions. Meanwhile, experiments such as crystal violet staining demonstrated the excellent anti-biofilm properties of NPDCDs_1_. In summary, this study confirms that the size of carbon dots is a key factor influencing their surface chemistry, optical properties, biocompatibility, and antibacterial activity. As the particle size decreases, surface oxygen-containing functional groups increase, and the fluorescence emission undergoes a red-shift. In terms of biological activity, the largest size fraction exhibits excellent biocompatibility and strong antibacterial activity, whereas the smaller size fractions show cytotoxicity but weaker antibacterial performance. These findings provide important experimental evidence for the design of size-engineered antibacterial carbon dots. However, it is important to note that, as size and surface chemistry co-varied in this system, the corresponding contributions were not fully disentangled. Future work should systematically decouple these two variables to further elucidate the molecular mechanisms of the synergistic effects.

## Figures and Tables

**Table 1:** The proportions of C, N, and O elements in each component of the NPDCDs were analyzed by XPS.

Samples	Elements	Atomic Ratio (%)
NPDCDs1	C	68.25
	N	9.67
	O	22.09
NPDCDs2	C	60.46
	N	16.83
	O	22.71
NPDCDs3	C	71.65
	N	12.34
	O	16.01
NPDCDs4	C	55.47
	N	9.72
	O	34.81

**Table 2:** Chemical forms of carbon-containing compounds identified from C 1s spectrum fitting results.

Samples	Peaks	Oxygen Form	Atomic Ratio (%)
NPDCDs_1_	1	C-C	30.22
	2	C=C	8.45
	3	C-O	23.65
	4	C=O	5.93
NPDCDs_2_	1	C-C	35.80
	2	C=C	17.13
	3	C-O	6.75
	4	C=O	2.81
NPDCDs_3_	1	C-C	32.35
	2	C=C	27.74
	3	C-O	7.34
	4	C=O	5.13
NPDCDs_4_	1	C-C	19.30
	2	C=C	9.48
	3	C-O	9.74
	4	C=O	5.06

**Table 3:** Chemical forms of nitrogen-containing compounds identified from N 1s spectrum fitting results.

Samples	Peaks	Oxygen Form	Atomic Ratio (%)
NPDCDs_1_	1	Pyridinic N	2.72
	2	Graphitic N	6.74
NPDCDs_2_	1	Pyridinic N	3.19
	2	Graphitic N	12.94
NPDCDs_3_	1	Pyridinic N	3.42
	2	Graphitic N	9.06
NPDCDs_4_	1	Pyridinic N	7.93
	2	Graphitic N	12.52

**Table 4:** Chemical forms of oxygen-containing compounds identified from O 1s spectrum fitting results.

Samples	Peaks	Oxygen Form	Atomic Ratio (%)
NPDCDs_1_	1	C=O	4.32
	2	C-O	17.97
NPDCDs_2_	1	C=O	8.13
	2	C-O	13.25
NPDCDs_3_	1	C=O	5.13
	2	C-O	10.97
NPDCDs_4_	1	C=O	10.09
	2	C-O	25.86

**Table 5:** Precursors used for the synthesis of oPDCDs, CCDs, PhCDs, and PEICDs.

CDs	Materials
oPDCDs	o-phenylenediamine, polyethyleneimine, sodium citrate, 2,3-diaminophenazine
CCDs	4-nitro-o-phenylenediamine, polyethyleneimine, sodium citrate
PhCDs	Polyethyleneimine, 2,3-diaminophenazine
PEICDs	4-nitro-o-phenylenediamine, polyethyleneimine

## Data Availability

The original contributions presented in this study are included in the article/Supplementary Material. Further inquiries can be directed to the corresponding authors.

## Supplementary Materials

The following supporting information can be downloaded at: https://www.mdpi.com/article/10.3390/ijms27104159/s1.
