# Supplementary material for: Fabrication of Size-Controlled Carbon Dots with Biofilm-Disrupting Activity for Antibacterial Applications
Source: Int J Mol Sci. 2026 May 7;27(10):4159. doi: 10.3390/ijms27104159 (PMC13206887; doi:10.3390/ijms27104159)
Supplement: Supplementary file 1 [file ijms-27-04159-s001.zip › ijms-4207542-supplementary.pdf]

Fabrication of Size-controlled Carbon Dots with Biofilm-disrupting Activity for Antibacterial Applications

Yu-Xin Qian [1#] , Ming Yu [2#] , Ze-Kun Chen [2,3] , Yue Shen [2] , Lei Tang [1*] , Ke-Wu Zeng [2,3*] , and Peng-Fei Tu [2]

[1]School of Pharmaceutical Sciences, Guizhou Medical University, Guiyang 550014, China;

[2]State Key Laboratory of Natural and Biomimetic Drugs, School of Pharmaceutical Sciences, Peking University, Beijing 100191, China;

[3]Department of Integration of Chinese and Western Medicine, School of Basic Medical Sciences, Peking University, Beijing 100191, China.

Supplementary Information

Fig. S1. XPS survey scans and high-resolution spectra of NPDCDs (cited in Section 2.1).

Fig. S2. Zeta potentials of each NPDCDs fraction in PBS solutions at pH 3-9(cited in Section 2.2).

Fig. S3. Cell viability after co-incubation of each NPDCDs fraction with 293T and HepG2 cells (cited in Section 2.4).

Fig. S4. The minimum inhibitory concentration of $NPDCDs_1$ (cited in Section 2.5).

Table S1. The 16S rRNA sequencing sequences of the bacterial strains used.

Table S2. The lacY gene sequences of the bacterial strains used.

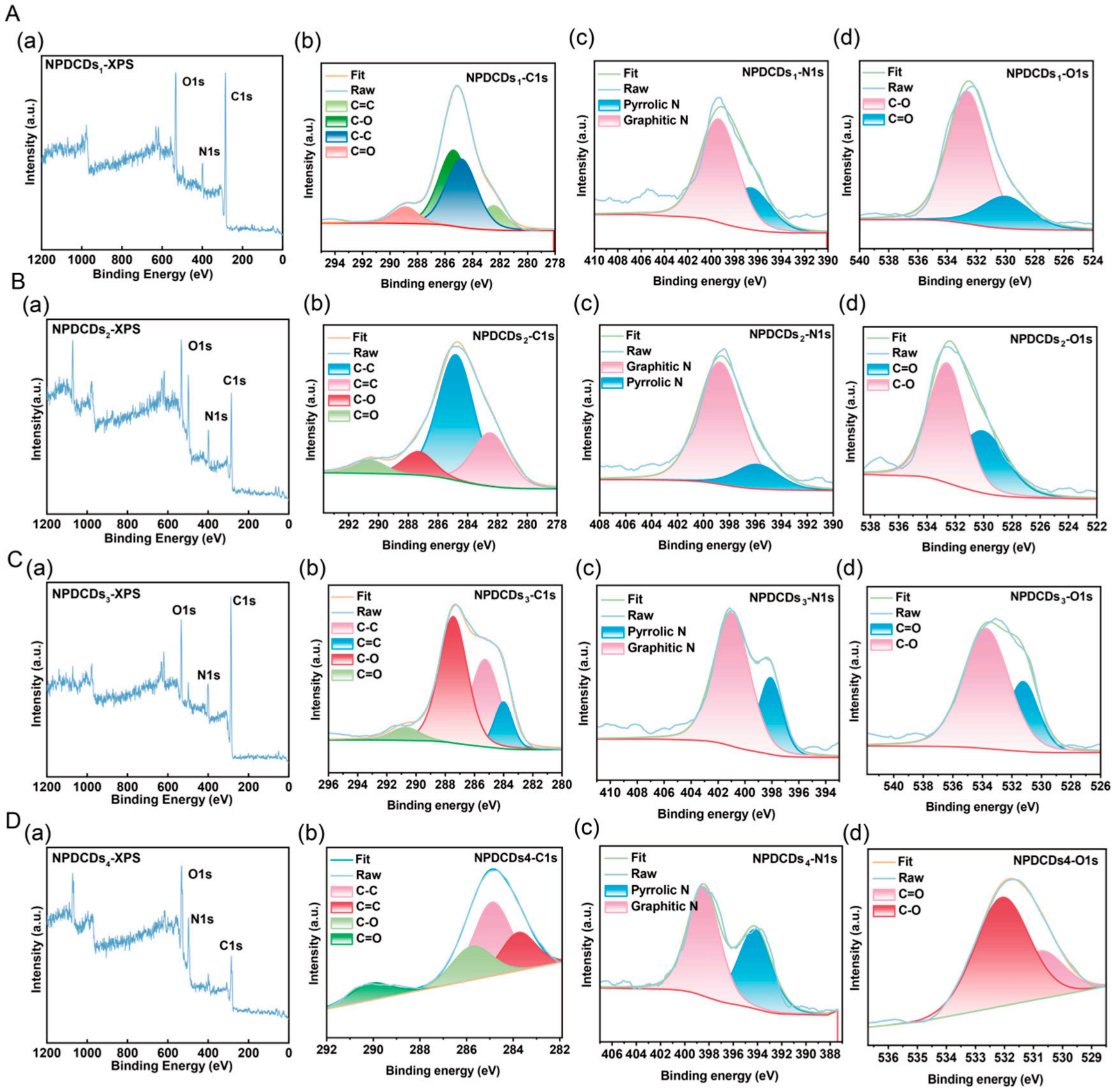

Fig. S1. XPS survey scans and high-resolution spectra of NPDCDs. (A) Survey scan and high-resolution spectra of $NPDCDs_1$; (B) Survey scan and high-resolution spectra of $NPDCDs_2$; (C) Survey scan and high-resolution spectra of $NPDCDs_3$; (D) Survey scan and high-resolution spectra of $NPDCDs_4$.

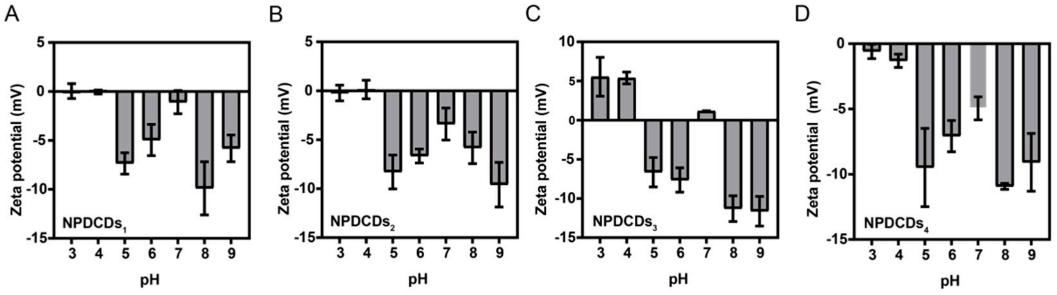

Fig. S2. Zeta potentials of each NPDCDs fraction in PBS solutions at pH 3-9.

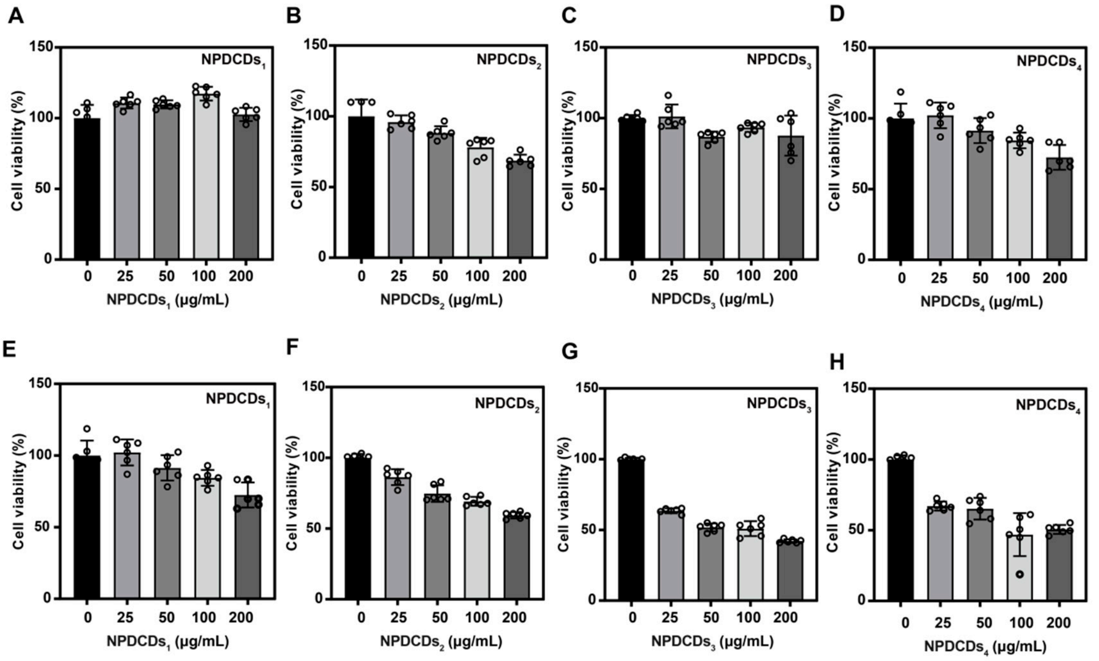

Fig. S3. Cell viability after co-incubation of each NPDCDs fraction with 293T and HepG2 cells. (A-D) Viability of 293T cells following 24 h exposure to NPDCDs; (E-H) Viability of HepG2 cells following 24 h exposure to NPDCDs.

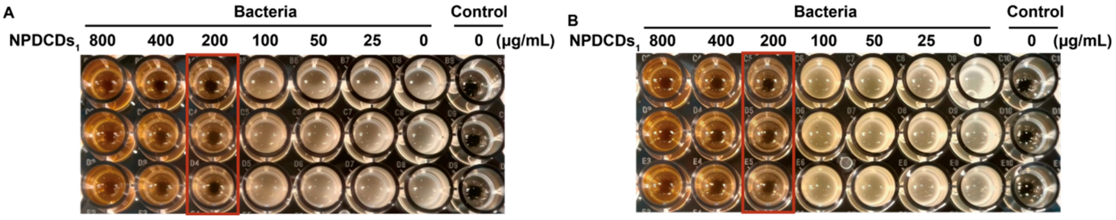

Fig. S4. The minimum inhibitory concentration of NPDCDs$_1$.

Table S1. The 16S rRNA sequencing sequences of the bacterial strains used.

Identification of bacterial species	Strain sequence
	CTACACATGCAGTCGACGGTAACAGGAAGCAGCTTGCTGCTTNGCTGAC GAGTGGCGGACGGGTGAGTAATGTCTGGGAAACTGCCTGATGGAGGGG GATAACTACTGGAAACGGTAGCTAATACCGCATAACGTCGCAAGACCAAA GAGGGGGACCTTCGGGCCTCTTGCCATCGGATGTGCCCAGATGGGATTA

Escherichia coli strain	GCTAGTAGGTGGGGTAACGGCTCACCTAGGCGACGATCCCTAGCTGGTC<br>TGAGAGGATGACCAGCCACACTGGAACTGAGACACGGTCCAGACTCCTA<br>CGGGAGGCAGCAGTGGGGAATATTGCACAATGGGCGCAAGCCTGATGC<br>AGCCATGCCGCGTGTATGAAGAAGGCCTTCGGGTTGTAAAGTACTTTCAG<br>CGGGGAGGAAGGGAGTAAAGTTAATACCTTTGCTCATTGACGTTACCCGC<br>AGAAGAAGCACCGGCTAACTCCGTGCCAGCAGCCGCGGTAATACGGAG<br>GGTGCAAGCGTTAATCGGAATTACTGGGCGTAAAGCGCACGCAGGCGGT<br>TTGTTAAGTCAGATGTGAAATCCCCGGGCTCAACCTGGGAACTGCATCTG<br>ATACTGGCAAGCTTGAGTCTCGTAGAGGGGGGTAGAATTCCAGGTGTAG<br>CGGTGAAATGCGTAGAGATCTGGAGGAATACCGGTGGCGAAGGCGGCC<br>CCCTGGACGAAGACTGACGCTCAGGTGCGAAAGCGTGGGGAGCAAACA<br>GGATTAGATACCCTGGTAGTCCACGCCGTAAACGATGTCGACTTGGAGGT<br>TGTGCCCTTGAGGCGTGGCTTCCGGAGCTAACGCGTTAAGTCGACCGCC<br>TGGGGAGTACGGCCGCAAGGTTAAAACTCAAATGAATTGACGGGGGCCC<br>GCACAAGCGGTGGAGCATGTGGTTTAATTCGATGCAACGCGAAGAACCT<br>TACCTGGTCTTGACATCCACNGAANTTTNCAGAGATGNNNNNGTGCCTTC<br>GGGAACNGTGAGACAGGTGCTGCATGGCTGTCGTCAGCTCGTGTTGTG<br>AAATGTTGGGTTAAGTCCCGCAACGAGCGCAACCCTTATCCTTTGTTGCC<br>AGCGGTCCGGCCGGGAACTCAAAGGAGACTGCCAGTGATAAACTGGAG<br>GAAGGTGGGGATGACGTCAAGTCATCATGGCCCTTACGACCAGGGCTAC<br>ACACGTGCTACAATGGCGCATACAAAGAGAAGCGACCTCGCGAGAGCAA<br>GCGGACCTCATAAAGTGCGTCGTAGTCCGGATTGGAGTCTGCAACTCGA<br>CTCCATGAAGTCGGAATCGCTAGTAATCGTGGATCAGAATGCCACGGTGA<br>ATACGTTCCCGGGCCTTGTACACACCGCCCGTCACACCATGGGAGTGGG<br>TTGCAAAAGAAGTAGGTAGCTTAACCTTCGGGAGGGCGCTACCACT
Staphylococcus aureus strain	TCGAGCGAACGGACGAGAAGCTTGCTTCTCTGATGTTAGCGGCGGACG<br>GGTGAGTAACACGTGGATAACCTACCTATAAGACTGGGATAACTTCGGGA<br>AACCGGAGCTAATACCGGATAATATTTTGAACCGCATGGTTCAAAAGTGAA<br>AGACGGTCTTGCTGTCACTTATAGATGGATCCGCGCTGCATTAGCTAGTT<br>GGTAAGGTAACGGCTTACCAAGGCAACGATGCATAGCCGACCTGAGAGG<br>GTGATCGGCCACACTGGAACTGAGACACGGTCCAGACTCCTACGGGAG<br>GCAGCAGTAGGGAATCTTCCGCAATGGGCGAAAGCCTGACGGAGCAAC<br>GCCGCGTGAGTGATGAAGGTCTTCGGATCGTAAAACTCTGTTATTAGGGA<br>AGAACATATGTGTAAGTAACTGTGCACATCTTGACGGTACCTAATCAGAAA<br>GCCACGGCTAACTACGTGCCAGCAGCCGCGGTAATACGTAGGTGGCAAG<br>CGTTATCCGGAATTATTGGGCGTAAAGCGCGCGTAGGCGGTTTTTTAAGT<br>CTGATGTGAAAGCCCACGGCTCAACCGTGGAGGGTCATTGGAAACTGGA<br>AAACTTGAGTGCAGAAGAGGAAAGTGGAATTCCATGTGTAGCGGTGAAAT<br>GCGCAGAGATATGGAGGAACACCAGTGGCGAAGGCGACTTTCTGGTCTG<br>TAACTGACGCTGATGTGCGAAAGCGTGGGGATCAAACAGGATTAGATACC<br>CTGGTAGTCCACGCCGTAAACGATGAGTGCTAAGTGTTAGGGGGTTTCC<br>GCCCCTTAGTGCTGCAGCTAACGCATTAAGCACTCCGCCTGGGGAGTAC<br>GACCGCAAGGTTGAAACTCAAAGGAATTGACGGGGACCCGCACAAGCG<br>GTGGAGCATGTGGTTTAATTCGAAGCAACGCGAAGAACCTTACCAAATCT

	TGACATCCTTTGACAACTCTAGAGATAGAGCNTTCCCCTTCGGGGGACAA AGTGACAGGTGGTGCATGGTTGTCGTCAGCTCGTGTCGTGAGATGTTGG GTTAAGTCCCGCAACGAGCGCAACCCTTAAGCTTAGTTGCCATCATTAAG TTGGGCACTCTAAGTTGACTGCCGGTGACAAACCGGAGGAAGGTGGGG ATGACGTCAAATCATCATGCCCCTTATGATTTGGGCTACACACGTGCTACA ATGGACAATACAAAGGGCAGCGAAACCGCGAGGTCAAGCAAATCCCATA AAGTTGTTCTCAGTTCGGATTGTAGTCTGCAACTCGACTACATGAAGCTG GAATCGCTAGTAATCGTAGATCAGCATGCTACGGTGAATACGTTCCCGGG TCTTGTACACACCGCCCGTCACACCACGAGAGTTTGTAACACCCGAAGC CGGTGGAGTAACCTTTTAGGAGCT

Table S2. The lacY gene sequences of the bacterial strains used.

Identification of bacterial species	Strain sequence
Escherichia coli strain	TACCAGACCCCCACACCAGATAAGCGCCCTGGAAGCCGATGCTTTCATAC ATATTGCCCGCCAGTACAGACATAAAAATCATCGCCAGTTGCTTAAAGAAG CAGAAACAGACCAGATAAATCGTCGCTGAAAAACGCACTTCAAACTGGCT GGTAATATATTTAAAGCAGCCTACCAGCAGGAACGGTACTTCAAACATATG CAGCGTTTTCAGAATAACTACTTCCAGCGCTGAGGTGGCGAACGATGAG CCAATAATACGTACAGACATAATAGTGCCAGCCAGCAGCAGGGCGTTTTT CCCACCGATGCGATTAATGATCAGTGGCGCAAAGAACATAATCGAGGCGT TAAGTAATTCGCCCATTGTCGTTACGTAGCCAAATACCCGCGTACCCTGTT CACCGGTAGCAAAGAACGAAGTAAAGAAATTAGCAAACTGTTGGTCAAAA ACACGGAAAGGTGCA
